# Reducing the Pill Burden: Immunosuppressant Adherence and Safety after Conversion from a Twice-Daily (IR-Tac) to a Novel Once-Daily (LCP-Tac) Tacrolimus Formulation in 161 Liver Transplant Patients

**DOI:** 10.3390/biomedicines10020272

**Published:** 2022-01-26

**Authors:** Max M. Maurer, Marius Ibach, Julius Plewe, Axel Winter, Paul Ritschl, Brigitta Globke, Robert Öllinger, Georg Lurje, Wenzel Schöning, Johann Pratschke, Dennis Eurich

**Affiliations:** 1 Department of Surgery, Experimental Surgery, Campus Charité Mitte, | Campus Virchow Klinikum, Charité–Universitätsmedizin Berlin, Freie Universität Berlin, Humboldt-Universität zu Berlin, Charitéplatz 1, 10117 Berlin, Germany; Marius.Ibach@charite.de (M.I.); Julius-Maximilian.Plewe@charite.de (J.P.); Axel.Winter@charite.de (A.W.); Paul.Ritschl@charite.de (P.R.); Brigitta.Globke@charite.de (B.G.); Robert.Oellinger@charite.de (R.Ö.); Georg.Lurje@charite.de (G.L.); Wenzel.Schoening@charite.de (W.S.); Johann.Pratschke@charite.de (J.P.); Dennis.Eurich@charite.de (D.E.); 2 BIH Charité Clinician Scientist Program, Berlin Institute of Health (BIH), 13353 Berlin, Germany

**Keywords:** immunosuppressant adherence, immunosuppressant efficacy, tacrolimus, liver transplantation

## Abstract

Non-adherence to immunosuppressant therapy reduces long-term graft and patient survival after solid organ transplantation. The objective of this 24-month prospective study was to determine adherence, efficacy and safety after conversion of stable liver transplant (LT) recipients from a standard twice-daily immediate release Tacrolimus (IR-Tac) to a novel once-daily life cycle pharma Tacrolimus (LCP-Tac) formulation. We converted a total of 161 LT patients at baseline, collecting Tacrolimus trough levels, laboratories, physical examination data and the BAASIS^©^ questionnaire for self-reported adherence to immunosuppression at regular intervals. With 134 participants completing the study period (17% dropouts), the overall adherence to the BAASIS^©^ increased by 57% until month 24 compared to baseline (51% vs. 80%). Patients who required only a morning dose of their concomitant medications reported the largest improvement in adherence after conversion. The intra-patient variability (IPV) of consecutive Tacrolimus trough levels after conversion did not change significantly compared to pre-conversion levels. Despite reducing the daily dose by 30% at baseline as recommended by the manufacturer, Tac-trough levels remained stable, reflected by an increase in the concentration-dose (C/D) ratio. No episodes of graft rejection or loss occurred. Our data suggest that the use of LCP-Tac in liver transplant patients is safe and can increase adherence to immunosuppression compared to conventional IR-Tac.

## 1. Introduction

The calcineurin inhibitor (CNI) Tacrolimus (Tac) has remained the standard immunosuppressant after solid organ transplantation for more than two decades [1,2,3]. Apart from its high efficacy in preventing graft rejection, Tac exhibits a narrow therapeutic index with considerable variability in systemic drug exposure [4,5]. Patients, therefore, depend on therapeutic drug monitoring via regular Tac-trough concentration measurements and may require dose adjustments to minimize toxicity without compromising efficacy [6]. While underdosing Tac may promote graft rejection and ultimately graft failure, overdosing and high Tac serum levels typically increase the risk for renal injury, de novo malignancies, neurotoxic side effects, opportunistic infections, and metabolic dysregulation [7,8,9,10]. In patients with high Tac doses, elevated peak concentrations are associated with increased CNI-toxicity independently of 24-h exposure (AUC—area under the curve) [11]. The current standard of care, twice-daily immediate-release Tacrolimus (IR-Tac) exhibits a relatively low bioavailability due to poor water solubility, a marked pre-systemic metabolism by CYP3A in the enterocytes, and extensive first-pass metabolism [4]. In addition to Tac blood level management being complicated by highly individual pharmacokinetic characteristics and the low bioavailability of IR-Tac, patients must take the formula twice per day. However, multiple doses per day increase the risk of poor adherence [12]. In “The Swiss Transplant Cohort Study”, De Geest et al. (2013) showed that medication nonadherence negatively affects both graft and patient survival after liver transplantation [13]. In an analysis of Scottish registry data, poor adherence was associated with up to 10% of deaths of liver transplanted patients and may have caused chronic rejection in 30% of re-transplantations [14]. Hence, simplification of the daily medication regimen may improve drug intake and long-term outcomes after liver transplantation [12]: In a group of 125 stable liver transplant recipients converted from twice daily IR-Tac to a once-daily extended-release Tacrolimus (ER-Tac) formulation, medication adherence significantly improved over 12 months (30% to 64%; *p* < 0.001), without compromising efficacy or safety [15].

Life cycle pharma Tacrolimus (LCP-Tac; *Envarsus*^®^) is a novel once-daily tablet formulation that uses a “MeltDose drug delivery technology”, which effectively dissolves Tac into single drug molecules to improve enteric uptake. This increases oral bioavailability and facilitates controlled release. Pharmacokinetic head-to-head studies show that LCP-Tac exhibits a longer time to peak (T_max_) and lower maximum concentrations (C_max_) compared to both IR-Tac and ER-Tac [16]. In an open, multicentric and prospective study in 57 stable liver transplant recipients, the greater bioavailability of LCP-Tac allowed for once-daily dosing with comparable systemic exposure (AUC) and trough levels at 30% less total daily dosage (TDD) compared to IR-Tac. In addition, LCP-Tac resulted in a more consistent Tac exposure over the course of 24 h characterized by reduced peak-to-trough fluctuations versus IR-Tac [17].

So far, adherence data of liver transplant patients are only available for conversion from IR-Tac to ER-Tac. There is also only limited data available on the long-term tolerability and effectiveness of LCP-Tac. The aim of the present study was therefore to assess adherence to immunosuppressive medication in a cohort of stable liver transplant recipients after conversion of their medication regimen from a twice-daily IR-Tac formulation to the novel once-daily LCP-Tac. In addition, we assessed the long-term tolerability and effectiveness of LCP-Tac over a two-year-study period.

## 2. Materials and Methods

### 2.1. Study Design

**Figure 1:**
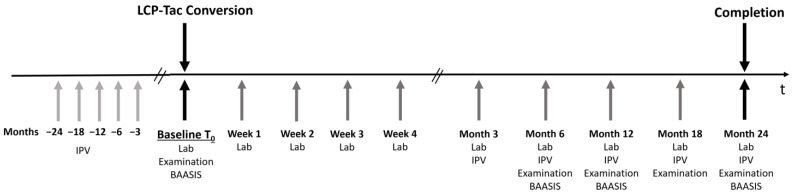
Study design depicting the measurements taken at each time point. IPV values prior to conversion were retrospectively retrieved from patients’ medical record files. Lab = laboratory blood tests, examination = physical examination. BAASIS = Basel Assessment of Adherence Scale to Immunosuppressives.

This was a prospective non-interventional, non-randomized, monocentric, single-arm, investigator-initiated observational study, performed at the Department of Surgery, Charité Campus Mitte|Campus Virchow Klinikum, Charité-Universitätsmedizin Berlin, Germany. The clinical condition of each patient was monitored as part of our routine liver transplant follow-up care program at 9 time points after conversion: weeks 1, 2, 3, and 4, and months 3, 6, 12, 18 and 24 (Figure 1). The routine laboratory parameters investigated at these visits contained electrolytes, liver and renal function, diabetes, and hypertriglyceridemia/hypercholesterolemia. In addition, non-routine evaluations of HEENT (head, eyes, ears, nose, and throat) and a standard neurologic exam (mental status, cranial nerves, reflexes, sensory and motor system, gait) were performed at baseline and months 6, 12, 18, and 24. Routine biopsies were performed in accordance with our regular liver transplant follow-up care program. Self-reported adherence with immunosuppressive treatment was assessed at baseline and at months 6, 12, and 24 after conversion using the ‘‘Basel Assessment of Adherence Scale to Immunosuppressives’’ (BAASIS^©^) including a four-item validated questionnaire and a Visual Analog Scale (VAS) as previously published [18]. The four separate questions participants get orally asked by the investigator are: over the past four weeks, (1) “did you miss a single dose?”, (2) “did you miss several doses?”, (3) “did you delay intake?”, and (4) “did you reduce dosage by yourself?”. Non-adherence of a patient is defined as answering at least one of the questions with yes. As of the VAS, patients are asked to self-rate their overall intake accuracy by marking a point on a paper-based, scaled line reaching from 0 (no dose taken) over 100 (each dose taken at the correct time ± 2 h) to 110 (more doses taken than prescribed).

Tac trough levels were measured at each time point. For IPV calculation, the values at months 3, 6, 12, 18 and 24 were used. For pre-conversion comparison, IPV data were derived retrospectively from patients’ medical records at the corresponding time intervals prior to conversion (month 3, 6, 12, 18 and 24 before study entry).

Any incidence and details of adverse events occurring during the study were recorded.

### 2.2. Inclusion and Exclusion Criteria

Eligible participants were stable adult liver transplant recipients treated with IR-Tac and patients recently (≤2 weeks before inclusion in the study) converted to LCP-Tac with available BAASIS^©^ assessment data at baseline. Exclusion criteria included any Tac dose adjustment during the prior 6 weeks before conversion, HCV RNA positivity, and the occurrence of acute cellular rejection within the last 6 months prior to study inclusion. The study was approved by the Charité’s Ethics Committee (EA2/027/16). Written consent was obtained from all patients for pseudonymized data collection and analysis. The study was funded by the company Chiesi GmbH. The funding played no role in the study design, the conduct of the study, the analysis and interpretation of the data, the preparation of the manuscript and the decision to publish the results.

### 2.3. Primary and Secondary Objectives

The primary objective was to detect changes in adherence to immunosuppressive medication at 6, 12 and 24 months in liver transplant patients who were recently converted from IR-Tac to LCP-Tac within our regular liver transplant patient program. We measured adherence using the BAASIS^©^ questionnaire as primary outcome and the Tac-trough level IPV as a surrogate parameter of medication intake.

### 2.4. Statistical Analysis

Based on an expected change in the percentage of adherent patients on the BAASIS^©^ by 10% and a dropout rate of 10%, a study sample of 148 patients was calculated to have 80% power to detect a difference at α = 0.05 (two-sided) using McNemar’s test, with a target enrollment of 165. Since we required an interval of 24 months for the pre-conversion IPV calculation and a minimum time since transplantation of 6 months for collection of the first Tac through level, only patients with an interval of >30 months since transplantation were considered for the IPV analysis (*n* = 85).

For analysis, McNemar’s test was used for univariate and a binary regression model for multivariate testing. The coefficient of variation for IPV analysis was calculated using the SD/mean of consecutive Tac levels. For IPV comparison, the Pitman Morgan test was applied to compare variability.

All data analysis was performed using statistical software IBM SPSS Statistics software Version 26 (SPSS Inc., Chicago, IL, USA).

## 3. Results

### 3.1. Patient Baseline Characteristics

**Figure 2:**
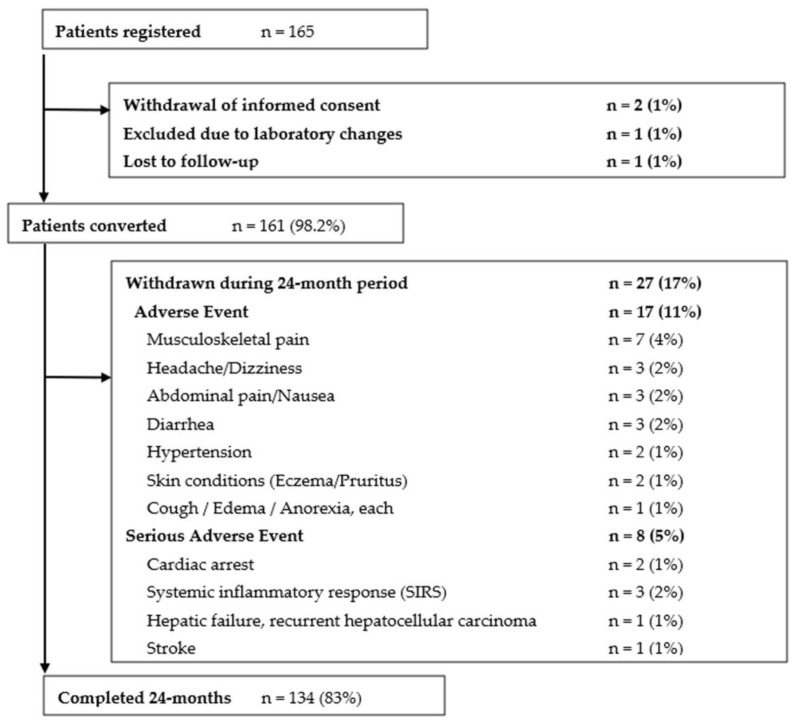
Patient disposition.

Between October 2016 and March 2018, 165 patients were screened for eligibility criteria. 161 patients (98%) received the study drug, and 134 patients (83%) completed the 24-month follow-up period (Figure 2). The median age at study entry was 58 (range: 21–85) years and around half of the patients (49.7%) were female. On average, the interval between transplantation and study inclusion was 7.5 years. Alcoholic liver disease (20%), hepatocellular carcinoma (20%), and autoimmune hepatitis (19%) were the most frequent indications for transplantation. Within the baseline cohort, more than half of the patients (54.5%) suffered from arterial hypertension, and 44.2% had a history of dyslipidemia or diabetes. Notably, 59% of the patients were treated with a regimen encompassing a second or third immunosuppressant in addition to Tac (Table 1).

### 3.2. Overall Adherence to Immunosuppressive Medication

Conversion from IR-Tac to LCP-Tac led to a significant increase in patient adherence (Table 2): At baseline, only half of the patients (51%) adhered to their Tac-based treatment protocol–15% reported unreliability in taking every dose, and 43% frequently delayed their medication for more than two hours. 24 months after conversion, the rate of adherent patients who took their medication correctly both quantitatively and qualitatively had increased to 80%, reflecting an improvement by 57%. In fact, the proportion of patients who missed any Tac-dose declined from 15% to 2% as decreased the rate of patients delaying their medication from 43% to 20% indicating a significant improvement in adherence for both aspects. In line with these results, the number of patients who self-assessed their medication behavior based on the VAS as “perfect adherence (100)” increased by 77%. When examining the IPV, however, neither the median coefficient of variation nor the standard deviation of consecutive Tac blood levels changed significantly compared with the variation during the two years under IR-Tac prior to conversion (Table 3).

### 3.3. Impact of Gender, Age, and Treatment Complexity on Patients’ Adherence

**Figure 3:**
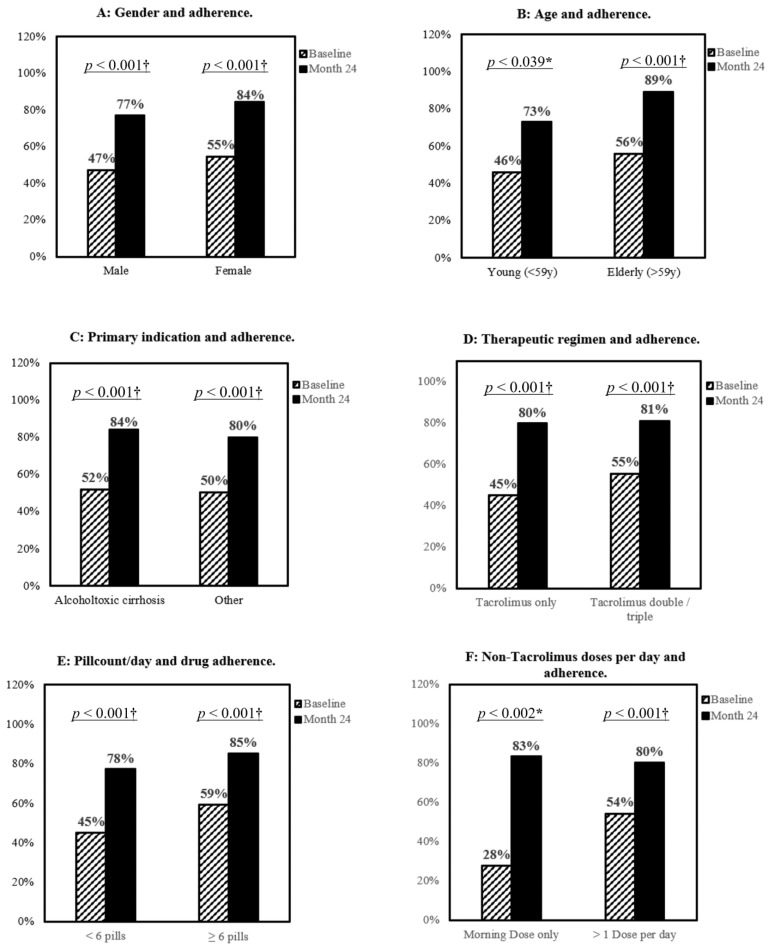
Evolution of overall adherence split by gender (**A**), age (**B**), primary indication (**C**), and therapeutic complexity (**D**–**F**), *n* = 134. * *p* = < 0.05, † *p* < 0.001, McNemar’s test.

Women reported higher adherence rates than men, both at baseline and month 24 after conversion. However, a significant improvement of 30% was observed for both sexes according to the BAASIS^©^questionnaire (Figure 3A). Considering age, patients older than 59 years tended to have a higher adherence already at baseline (56%), which further increased until month 24 (89%). Younger patients (<59 years) also improved significantly compared to baseline, but they did not catch up with the elderly (73% vs. 89%). The average number of concomitant medications at baseline was 6 ± 3 per day, ranging from one to 16 different pills. The mean number of medications per day did not affect Tac adherence as there was neither a significant difference between non-adherent (5.5 medications/day) and adherent (6.2 medications/day) patients at baseline nor 24 months after conversion (5.4/day vs. 6.0/day, *p* = 0.34). Likewise, the mean number of daily concomitant medication doses, e.g., in the morning, afternoon or evening, did not differ between adherent and non-adherent patients at baseline (2.0 vs. 1.9, *p* = 0.39) and 24 months after conversion (2.0 vs. 2.0, *p* = 0.79). However, the relative improvement in adherence for patients with a pill load <6 pills per day was greater (+33%) than for patients with a pill load ≥6 pills (26%) (Figure 3E).

Interestingly, patients with Tac-based monotherapy regimen reported a 10% lower adherence at baseline than patients with more complex therapy regimens. After 24 months, both patients under Tac-based monotherapy and under a Tac-based dual or triple immunosuppressive regimen achieved an improvement in adherence to 80% and 81%, respectively (Figure 3D). Notably, the largest improvement in adherence was observed for patients who only had to take their concomitant medications once in the morning in addition to twice daily IR-Tac, and who, since switching to LCP-Tac, only had to take Tac once in the morning (Figure 3F).

In a multivariate binary regression, old age was the only factor associated with adherence to immunosuppression on the BAASIS^©^ at month 24 (*p* = 0.2). In contrast, gender, pill and daily dose count as well as primary indication did not predict adherence in the multivariate model.

### 3.4. Pharmacokinetic Characteristic

According to the manufacturer, equivalent mean systemic Tac exposure (AUC) is achieved by using a 1:0.7 (mg:mg) ratio when converting IR-Tac formulations to LCP-Tac in stable transplant patients [19]. Therefore, the mean daily Tac dose was decreased from an average of 3.5 ± 1.6 mg IR-Tac at study entry to 2.4 ± 1.2 mg LCP-Tac during the first week, representing a relative dose reduction of 32% (Table 4). On day 7, the mean blood level remained stable at 5.5 ± 2.4 ng/mL, while the concentration dose ratio (C/D) increased by 65% during the same period–reflecting the modified absorption provided by LCP-Tac. To set patients near the lower limit of the target trough level range of 3–7 ng/mL, further dose modifications were applied during the study. At the time of completion, the mean daily intake of Tac was 1.6 ± 0.9 mg, representing a dose reduction of 51% compared to baseline. At the same time, the mean Tac blood concentration had decreased by 24% compared to baseline values (4.1 ± 1.9 vs. 5.4 ± 2.1 ng/mL, Supplementary Materials, Figure S1) corresponding to a mean C/D ratio increase by 82% (Table 4).

### 3.5. Efficacy and Adverse Events

Within the 24-month study period, the patient and graft survival were 97% and no episodes of allograft loss or rejection occurred. Five participants died during the course of the study: two patients deceased as a result of cardiac arrest 60 and 632 days post-conversion–both suffered from pre-existing cardiovascular comorbidities. Two patients died due to systemic inflammatory response syndrome (SIRS) due to pneumococcal pneumonia and mesenteric ischemia on day 64 and day 568, respectively. In one case, a relapse of hepatocellular was diagnosed within the first week after conversion leading to death on day 182. In addition, one patient required treatment for acute liver failure 18 months after conversion and was switched to ER-Tac during hospitalization. We classified this case as a serious adverse event leading to withdrawal because the patient did not receive LCP-Tac at the time of death.

27 patients (17%) deliberately withdrew from LCP-Tac and were reconverted to IR-Tac. Overall, dropouts were more likely to receive a Tac monotherapy prior to conversion. They were also slightly older than completers, more likely to be female, and about one year longer post-transplant–but these differences did not reach statistical significance (Table 1).

A total of 248 non-serious adverse events (AEs) were recorded over the course of the study period (Table 5). According to the Medical Dictionary for Regulatory Activities (MedRA) classification, gastrointestinal disorders, and nervous system/psychiatric disorders, followed by respiratory, thoracic, and mediastinal disorders were most frequent. Very common AEs (≥10%) were upper respiratory tract infections (17%), headache (16%), diarrhea (11%), and fatigue (11%), while common AEs (1–10%) included pruritus (9%), (self-reported) weight gain (9%), abdominal pain (8%), and musculoskeletal pain (7%). Musculoskeletal disorders were also reported as the overall most common AE that led to withdrawal. Serious AEs encompassed all events (*n* = 51) which led to death, hospitalization, or disability throughout the study period with infections, hepatobiliary and vascular disorders being the most frequent non-fatal serious AEs (Table 6).

As assessed by regular laboratory tests, hepatic, renal, hematologic, and metabolic parameters remained stable across the study period (Supplementary Materials, Table S1). Neither the plasma creatinine nor the eGFR did reveal any significant changes between baseline and 24 months post-conversion. This was also true when renal function was assessed separately for fast and slow Tac metabolizers divided by the median C/D ratio prior to conversion with both groups showing equally stable results (Supplementary Materials, Figure S2). Regarding metabolic parameters, a relative increase of 3% in HbA1c was observed between baseline and completion.

Finally, we observed an improvement in the occurrence of patient self-reported tremor–although we did not follow a standardized neurological assessment as part of the study protocol. 38% of participants stated to experience tremor at study entry of whom more than one third (39.2%) described an improvement at the time of completion: 21.5% reported a noticeable decrease of symptoms and 17.6% a complete remission 24 months after conversion. In contrast, two patients reported an increase in symptoms and two a new tremor onset.

## 4. Discussion

Posttransplant non-adherence to immunosuppressive regimens is associated with poor clinical outcomes and remains a common problem for patients, including kidney and liver transplant recipients [13,20,21]. Strategies to simplify the immunosuppressive regimen and reduce patients’ pill burden may improve adherence and thus long-term morbidity and mortality [15,22].

The present study comprises the first description of medication adherence in a cohort of stable liver transplant patients converted from conventional twice-daily IR-Tac to novel once-daily LCP-Tac. We observed an improvement in overall adherence by 57% at the time of completion. Older patients (>59 years) tended to adhere better to their medication than younger patients, while women reported higher adherence rates than men both at baseline and after two years which contrasts with observations from another cohort [20]. Surprisingly, a Tac-based monotherapy regime was associated with a lower adherence at baseline than a Tac-based dual or triple therapy regime. Strikingly, a therapy regimen that only included medications including LCP-Tac to be taken once in the morning after conversion exhibited the largest positive effect: the rate of perfect adherence almost tripled by month 24. In summary, taking the results of multivariate testing into account, the improvement in adherence to immunosuppression may be larger in the elderly as compared to younger patients–although this group also reported a significant improvement. This observation is in line with prior findings that have demonstrated that younger patients experience more difficulties in taking their medication as planned. In summary, practitioners may expect improved adherence irrespective of therapeutic regimen complexity, gender, and primary indication, while elderly patients may experience greater benefits than younger patients.

Prior studies suggest that adherence may increase with more frequent follow-up visits [23]. In our study, the number of visits corresponded to the regular course of our LT-patients follow-up framework except for the first month. Since we only performed the BAASIS^©^ questionnaire at 6, 12 and 24 months, we cannot determine whether the adherence rates would have been further improved by shorter follow-up intervals–but since most transplant centers cannot offer significantly more frequent visits, a potential effect may have limited clinical consequences.

The intra-patient variability in Tac exposure is defined as the fluctuation in Tac blood concentrations within one patient over a certain period without dose adjustments. The coefficient of variation is most often used to quantify the IPV [24]. In our study, consecutive Tac levels under LCP-Tac seemed to fluctuate to a degree comparable with IR-Tac prior to conversion, as we observed no significant changes in the coefficient of variation nor the standard deviation. This observation might derive from various reasons: First, IPV and the BAASIS^©^ do not assess congruent time periods and–considering the Tacrolimus half-life of approximately twelve hours–IPV is sensitive to the drug exposure during the previous 2–4 days only. In contrast, the BAASIS^©^ relates to adherence characteristics covering the past month and therefore includes a more extended interval. Second, given the impact of several co-factors such as nutrition, co-medications or delayed blood withdrawal during outpatient clinic visits on Tac-trough measurements, the IPV might generally be a less valid tool to determine adherence. Third, as of today, no studies have validated the IPV as a measure for nonadherence. In addition, current evidence suggests that the Tac IPV should be–if at all–receive less emphasis in liver transplantation compared with kidney transplantation [24].

The graft and patient survival were considerably high (97%) in our study. In addition, there were no episodes of allograft loss or rejection over the two-year study period. As the pharmacokinetic data revealed, the mean daily Tac dose declined by 32% among participants during the first week after conversion to LCP-Tac. Despite the reduced dose, the Tac target range of 3–7 ng/mL was maintained. 24 months after completion, the daily Tac dose was 1.6 mg representing a dose reduction of 51% in comparison to the baseline level and hence reflecting an 82% increase in the C/D ratio. Notably, a low Tac blood concentration to daily dose ratio (C/D ratio) reflects a high rate of Tac metabolism and is strongly associated with an increased risk of CNI-induced nephrotoxicity [25]. Thus, a higher C/D ratio after conversion to LCP-Tac could have a nephroprotective effect on the liver transplant recipient [26]. However, neither a significant improvement nor deterioration in eGFR was observed in fast or slow Tac metabolizers after 24 months compared to baseline. Therefore, we cannot conclude that the different pharmacokinetics of LCP-Tac translate into a different nephrotoxic profile compared to IR-Tac. If the natural annual GFR decrease of one ml/min is taken into account, the observed GFR stability can argue for an insignificantly lower nephrotoxicity of the new formulation.

In our study, 17% of participants who were switched to LCP-Tac did not complete the full 24 months period due to AEs with most dropouts being caused by non-serious AEs and within the first four weeks after conversion, possibly demonstrating a strong bond with their usual, previously used medication. Musculoskeletal disorders and headaches were overall the most common AEs leading to withdrawal. Clinical laboratory findings were minimal and did generally not require intervention. However, our experience seemed to affirm that the flatter pharmacokinetic profile of LCP-Tac might be beneficial in mitigating peak-related effects such as neurotoxicity [21,27]. Even though the study was not designed to provide a meaningful evaluation of CNI-induced neurologic side effects, a notable number of patients experiencing tremor at study entry reported an improvement or complete cessation of their symptoms.

Our observational study was limited by its monocentric nature and by the non-randomized design. We cannot rule out a selection bias in favor of study participants who tend to have a higher level of adherence. Patient selection was based on the stability of the course of disease and the likelihood of a permanent need for stable immunosuppression long term. However, the study was carried out as part of the routine follow-up procedure at our transplant center, so that it largely reflects the real-world clinical setting at a university hospital in Germany.

## 5. Conclusions

The present study indicates that stable liver transplant patients may safely be converted from IR- to LCP-Tac. Conversion seems to be associated with a good clinical outcome and no increase in rejection with long-term use. The once-daily dosing of LCP-Tac was associated with a significant improvement in adherence compared to IR-Tac, and may have contributed to a decrease in CNI-induced tremor rates.

## Figures and Tables

**Table 1:** Descriptive characteristics of the study group at baseline, means ± SD, median (range) or N (%).

Characteristic	Completers	Non-Completers
**N**	134	31
**Female**	64 (48%)	18 (58%)
**Age at study entry, years**	55 ± 13	56 ± 13
**Time since LT, months**	91 ± 85	101 ± 87
Median (Range)	58 (4–336)	62 (5–312)
<1 year	14 (10%)	2 (7%)
2–5 years	57 (43%)	12 (39%)
5–10 years	19 (15%)	6 (19%)
>10 years	43 (32%)	9 (30%)
**BMI**	26 ± 5	25 ± 5
<18,5	5 (4%)	2 (6%)
18.5–25	53 (40%)	14 (45%)
25–30	47 (35%)	9 (29%)
>30	30 (22%)	6 (19%)
**Race**		
Caucasian	129 (96%)	31 (100%)
Other	5 (4%)	0 (0%)
**Primary indication**		
Autoimmune hepatitis	27 (20%)	3 (10%)
Hepatocellular carcinoma	26 (19%)	6 (19%)
Alcoholic liver disease	25 (19%)	7 (23%)
Acute liver failure	14 (10%)	2 (6%)
Chronic viral hepatitis C	9 (7%)	3 (10%)
Cryptogenic cirrhosis	8 (6%)	2 (6%)
Viral hepatitis B ^a^	8 (6%)	1 (3%)
Liver cysts	4 (3%)	0 (0%)
Nonalcoholic steatohepatitis	2 (1%)	3 (10%)
Wilson’s disease	2 (1%)	0 (0%)
Other	9 (7%)	4 (13%)
**Arterial hypertension**	73 (54%)	17 (55%)
Number of antihypertensive drugs		
1	43 (32%)	10 (32%)
2	22 (16%)	6 (20%)
3	6 (4%)	1 (3%)
4	1 (1%)	0 (0%)
**Dyslipidemia**	29 (22%)	10 (32%)
Statins/fibrates	17 (13%)	5 (16%)
**Diabetes**	25 (19%)	9 (29%)
Insulin/oral antidiabetics	20 (15%)	7 (23%)
**Tacrolimus-based immunosuppression**		
Plus mycophenolate mofetil	52 (39%)	7 (23%)
Plus prednisone	11 (8%)	2 (6%)
Plus everolimus	17 (13%)	2 (6%)
Tac-based monotherapy	60 (45%)	20 (65%)
Tac-based dual therapy regime	68 (51%)	11 (35%)
Tac-based triple therapy regime	6 (4%)	0 (0%)

^a^ one patient with simultaneous viral hepatitis D infection.

**Table 2:** ‘Basel assessment of Immunosuppressant Adherence Medication Scale’ (BAASIS^©^)–patients who completed 24-month period (*n* = 134), N (%) or mean ± SD.

Item	Baseline		Month 6		Month 12		Month 24	
**Questionnaire** Dose not taken	20	(15%)	8	(6%)	5	(4%)	3	(2%)
Consecutive doses not taken	1	(1%)	0	-	1	(1%)	0	-
Dose taken with >2 h delay	57	(43%)	41	(32%)	33	(25%)	26	(20%)
Dose reduced	0	-	0	-	1	(1%)	0	-
Overall non-adherence	66	(49%)	43	(33%)	35	(26%)	26	(20%)
**Visual Analog Scale**								
(0–100)	93 ± 11		97 ± 9		98 ± 6		98 ± 4	
Frequency optimal adherence ^a^	59	(44%)	85	(67%)	98	(72%)	102	(78%)

^a^ Number of patients who reported 100% adherence on the Visual Analog Scale.

**Table 3:** Effect of conversion to LCP-Tac on intra-patient variability of tacrolimus levels, *n* = 85.

	IR-Tac		LCP-Tac		
	*n* = 85		*n* = 85		
	Median	Range	Median	Range	*p*
Coefficient of variation C_0_/Dose	21%	4–85%	22%	4–70%	0.85
Standard deviation C_0_	1.1	0.2–5.0	1.2	0.2–3.6	0.68

**Table 4:** Tacrolimus pharmacokinetic parameters at baseline and after conversion to LCP-Tac, mean ± SD, min-max or *n* (%). Includes all available data.

	t = 0	Week 1	Month 1	Month 3	Month 6	Month 12	Month 18	Month 24
	*n* = 162	*n* = 143	*n* = 135	*n* = 141	*n* = 143	*n* = 140	*n* = 138	*n* = 134
Tacrolimus dose (mg/day) (min−max)	3.5 ± 1.6 (1.0–8.0)	2.4 ± 1.2 (0.8–6.0)	2.2 ± 1.1 (0.8–6.0)	2.1 ± 1.1 (0.8–5.0)	2.0 ± 1.0 (0.8–6.0)	1.8 ± 1.0 (0.8–6.0)	1.7 ± 1.0 (0.8–6.0)	1.6 ± 0.9 (0.8–6.0)
Tacrolimus blood concentration (ng/mL) (min-max)	5.4 ± 2.1 (2.4–12.7)	5.5 ± 2,4 (1.0–12.0)	4.9 ± 2.0 (1.6–10.6)	5.0 ± 2.1 (1.3–11.3)	4.7 ± 2.0 (1.6–10−0)	4.6 ± 2.1 (1–0−11.8)	4.3 ± 1.7 (1.0–9.3)	4.1 ± 1.9 (1.0–11.8)
Concentration/dose ratio (ng/mL per mg/day) (min-max)	1.7 ± 1.0 (0.4–5.8)	2.8 ± 1.7 (0.3–10.1)	2.7 ± 1.6 (0.5–10.6)	3.0 ± 1.8 (0.5–12.4)	3.0 ± 1.9 (0.6–13.2)	3.1 ± 2.0 (0.2–10.7)	3.1 ± 1.7 (0.5–8.3)	3.1 ± 1.7 (0.3–11.1)
% change in dosage to previous visit	-	−32%	−8%	−5%	−4%	−9%	−4%	−6%
% change in blood concentrations to previous visit	-	+3%	−11%	+2%	−6%	−2%	−7%	−5%
% change in concentration/dosage to previous visit	-	+65%	−4%	+11%	0%	+3%	+0%	+0%
Nr. patients with dose decrease (%)	-	136 (95%)	43 (26%)	30 (22%)	49 (36%)	34 (24%)	30 (22%)	16 (12%)
Nr. patients with dose increase (%)	-	4 (3%)	6 (4%)	4 (3%)	6 (4%)	6 (4%)	7 (5%)	4 (3%)
Nr. patients with no dose change (%)	-	3 (2%)	86 (52%)	101 (75%)	82 (60%)	99 (71%)	101 (73%)	109 (84%)

**Table 5:** Very common (<10%) and common (1–10%) adverse events during the study period, N (% of patients). MedDRA (Medical Dictionary for Regulatory Activities) preferred terms.

Adverse Events	Frequency	
**Respiratory, thoracic, and mediastinal disorders**		
Viral upper respiratory tract infection	28	(17%)
Cough	5	(3%)
**Nervous system and psychiatric disorders**		
Headache	25	(16%)
Dizziness	6	(4%)
Paresthesia	3	(2%)
Tremor	4	(2%)
Restlessness/Agitation	4	(2%)
Insomnia	2	(1%)
**Gastrointestinal disorders**		
Diarrhea	18	(11%)
Abdominal pain	13	(8%)
Nausea and vomiting symptoms	11	(7%)
Gastroenteritis	4	(2%)
Acid reflux (esophageal)	3	(2%)
**General disorders**		
Fatigue	18	(11%)
Asthenia	4	(2%)
Dry mouth	3	(2%)
Pyrexia	3	(2%)
Hyperhidrosis	2	(1%)
**Skin and subcutaneous disorders**		
Pruritus	15	(9%)
Eczema	8	(5%)
Basal cell carcinoma	3	(2%)
**Metabolism and nutrition disorders**		
Weight gain	14	(9%)
Edema, peripheral	3	(2%)
Weight loss	3	(2%)
**Renal and urinary disorders**		
Urinary tract infection	8	(5%)
**Infections and infestations**		
Herpes zoster	2	(1%)
**Vascular disorders**		
Hypertension worsened	10	(6%)
**Musculoskeletal and connective tissue disorders**		
Bone pain/Arthralgia	12	(7%)
Muscle cramps	3	(2%)
**Investigations**		
Hepatic enzyme increased	5	(3%)
Proteinuria	2	(1%)
**Cardiac disorders**		
Palpitations	2	(1%)
**Ear and labyrinth disorders**		
Tinnitus	2	(1%)

**Table 6:** Serious adverse events: includes all events which lead to death, hospitalization, or disability throughout the study period, N (% of patients). MedDRA (Medical Dictionary for Regulatory Activities) preferred terms.

Serious Adverse Events	Frequency	
*Death*		
Cardiac Arrest	2	(1%)
SIRS (Systemic inflammatory response syndrome)	2	(1%)
Hepatic failure–recurrent hepatocellular carcinoma	1	(1%)
*Non-fatal serious adverse events*		
**Infections and infestations**		
Abscess	3	(2%)
Pneumonia	3	(2%)
Urosepsis	2	(1%)
Hepatitis B/Epstein–Barr reactivation	2	(1%)
Herpes zoster	1	(1%)
**Hepatobiliary disorders**		
Bile duct stenosis	3	(2%)
Recurrent Hepatocellular carcinoma	2	(1%)
Cholangitis	2	(1%)
Hepatic failure	1	(1%)
Graft dysfunction	1	(1%)
Hepatomegaly	1	(1%)
**Vascular disorders**		
Thrombosis	3	(2%)
Pulmonary embolism	2	(1%)
**Renal and urinary disorders**		
Acute renal failure	2	(1%)
Urinary calculi	2	(1%)
**Respiratory, thoracic, and mediastinal disorders**		
Lung adenocarcinoma	2	(1%)
Pleural effusion	2	(1%)
**Gastrointestinal disorders**		
Gastrointestinal hemorrhage (gastric, small intestine)	2	(1%)
Diarrhea	2	(1%)
**Nervous system disorders**		
Stroke	1	(1%)
Transient ischemic attack	1	(1%)
**Psychiatric disorders**		
Hallucinations	1	(1%)
Paranoid schizophrenia	1	(1%)
**Other**		
Amyloidosis	1	(1%)
Anemia requiring transfusion	1	(1%)
Coronary artery disease	1	(1%)
Hyponatremia	1	(1%)
Post-transplant lymphoproliferative disorder (PTLD)	1	(1%)
Retinal detachment	1	(1%)
Rheumatoid arthritis	1	(1%)
Toxic epidermal necrolysis	1	(1%)

## Data Availability

Data can be provided upon request to Max-Magnus.Maurer@charite.de.

## Supplementary Materials

The following supporting information can be downloaded at: https://www.mdpi.com/article/10.3390/biomedicines10020272/s1, Figure S1: Individual Tacrolimus serum levels from all subjects throughout the 24 month study period.; Figure S2: Influence of metabolic group on tacrolimus pharmacokinetics (A-C) and renal function (D); Table S1: Median (range) of clinical and laboratory parameters at baseline, 12, and 24 months after conversion.
