# Supplementary material for: Reducing the Pill Burden: Immunosuppressant Adherence and Safety after Conversion from a Twice-Daily (IR-Tac) to a Novel Once-Daily (LCP-Tac) Tacrolimus Formulation in 161 Liver Transplant Patients
Source: Biomedicines. 2022 Jan 26;10(2):272. doi: 10.3390/biomedicines10020272 (PMC8869578; doi:10.3390/biomedicines10020272)
Supplement: Supplementary file 1 [file biomedicines-10-00272-s001.zip › biomedicines-1502142-supplementary.pdf]

Supplementary Materials

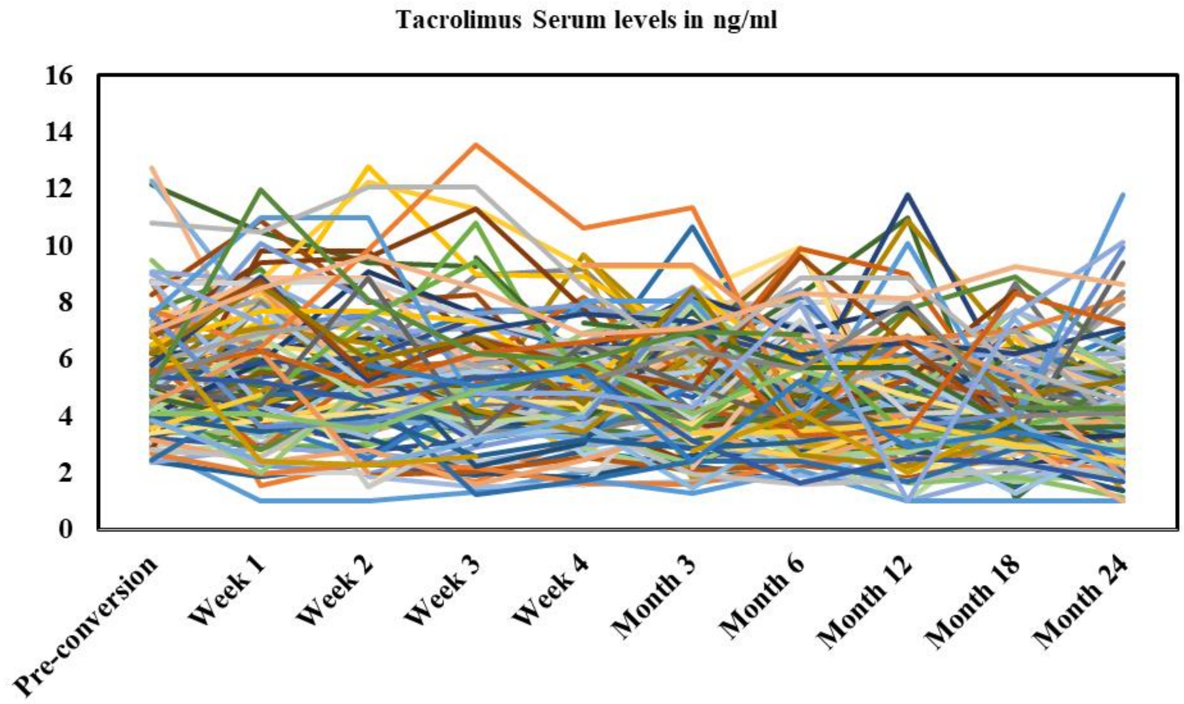

Figure S1. Individual Tacrolimus serum levels from all subjects throughout the 24 month study period.

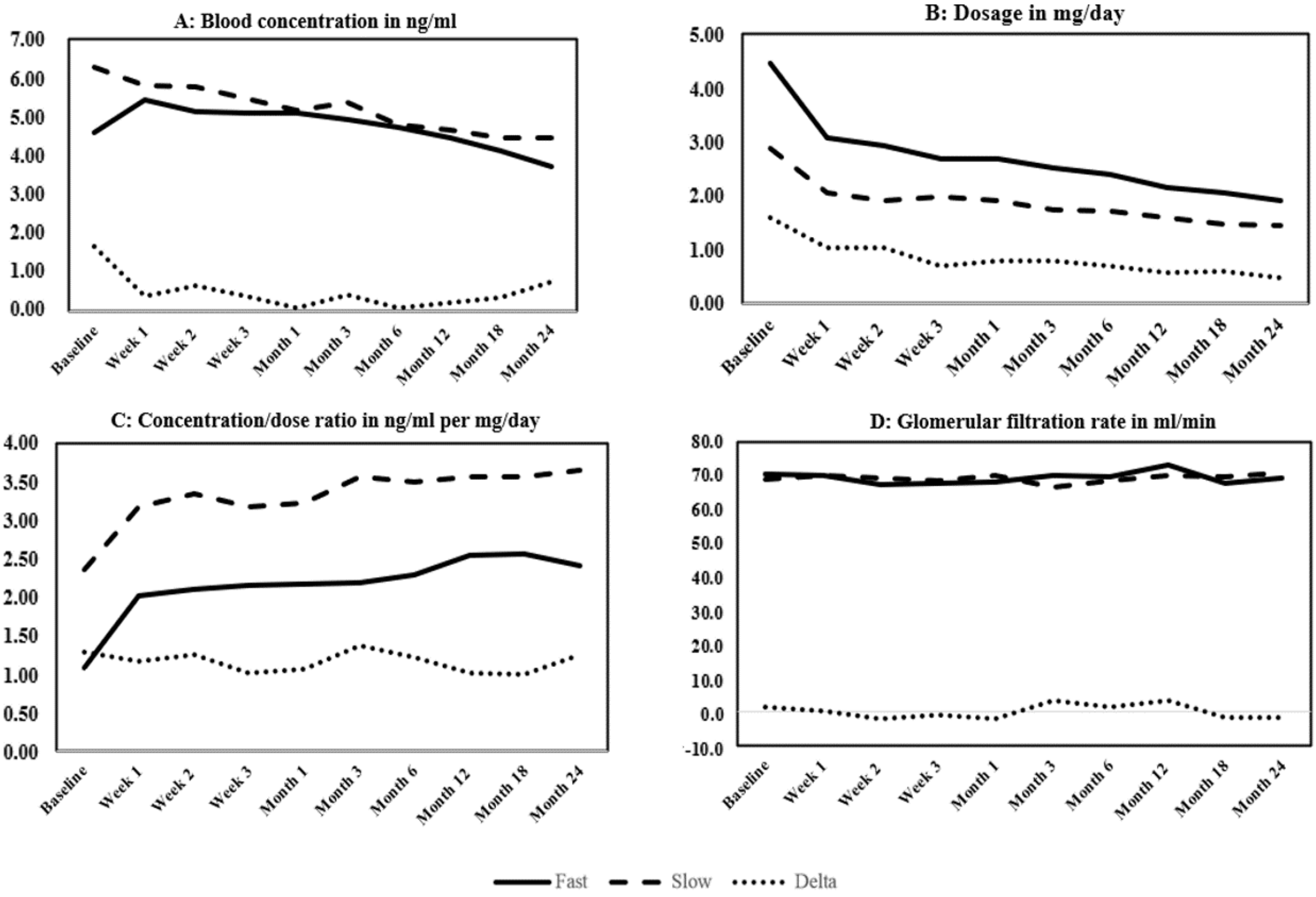

Figure S2. Influence of metabolic group on tacrolimus pharmacokinetics (A-C) and renal function (D), n = 124. Groups split by median C/D ratio at baseline into fast (C/D < 1.5) and slow (C/D > 1.5) metabolizers, Delta denotes difference between groups.

Table S1. Median (range) of clinical and laboratory parameters at baseline, 12, and 24 months after conversion. Missing values of dropouts were imputed using Last Observation Carried Forward or Next Observation Carried Backward, as appropriate.

Parameter. reference range	Baseline		Month 12		Month 24	
**Electrolytes**						
Potassium, 3.5 – 4.5 mmol/l	4.3	(3.4 – 5.9)	4.3	(3.0 – 6.0)	4.3	(3.1 – 6.0)
Calcium, 2.2 – 2.6 mmol/l	2.3	(2.1 – 2.6)	2.3	(2.0 – 2.7)	2.3	(2.1 – 2.6)
Magnesium, 0.66 – 0.99 mmol/l	0.8	(0.5 – 1.2)	0.8	(0.6 – 1.7)	0.8	(0.6 – 1.2)
Phosphate, 0.87 – 1.45 mmol/l	1.0	(0.5 – 1.5)	1.0	(0.6 – 2.1)	1.0	(0.6 – 2.1)
**Hepatic**						
AST, <50 U/l	25	(0 – 106)	26	(0 – 106)	25	(0 – 175)
ALT, <41 U/l	22	(0 – 209)	21	(0 - 209)	21	(0 - 140)
AP, 40-130 U/l	80	(42 – 457)	84	(42 - 652)	82	(42 - 638)
GGT, 8-61 U/l	24	(1 - 420)	25	(0 – 453)	27	(0 – 299)
Bilirubin, < 1.20 mg/dl	0.4	(0.2 – 2.5)	0.5	(0.2 – 2.6)	0.5	(0.2 – 1.7)
Albumin, 35 - 52 g/l	42	(31 – 50)	43	(31 - 69)	42	(22 - 74)
**Renal**						
Creatinine, 0.70-1.20 mg/dl	1.0	(0.5 – 4.7)	1.1	(0.5 – 5.5)	1.0	(0.5 – 6.4)
eGFR, ml/min/1.73m^2	68	(3.6 – 90)	68	(15 - 90)	69	(15 - 90)
Urea, 17- 48 mg/dl	39	(15 – 159)	34	(15 - 123)	36	(13 - 177)
**Hematological**						
Hemoglobin, 12.5 -17.2 g/dl	13.4	(7.2 – 17.4)	13.4	(5.9 - 17.9)	13.5	(5.9 - 18.1)
Leukocytes, 3.9 - 10.5 /nl	6.1	(1.6 – 18.5)	6.2	(2.0 - 23.8)	6.3	(2.0 – 23.8)
Thrombocytes, 150-370 /nl	196	(49 – 679)	199	(51 - 679)	203	(53 - 849)
**Metabolic & Cardiovascular**						
HbA1c, %	5.4	(3.5 – 13.5)	5.4	(3.5 – 9.9)	5.6	(3.3 – 11.0)
Fasting plasma glucose, mg/dl	101	(47 – 416)	97	(69 – 372)	99	(55 - 387)
Triglycerides, <200 mg/dl	104	(39 – 408)	105	(39 - 442)	106	(39 - 376)
Total Cholesterol, <200 mg/dl	176	(95 – 265)	176	(4 – 297)	175	(4 - 286)
HDL, >35 mg/dl	57	(21 – 106)	61	(26 - 232)	58	(12 – 106)
LDL, <130 mg/dl	111	(48 – 207)	111	(47 – 207)	107	(22 - 207)
Systolic blood pressure, <140 mmHg	137	(90 – 181)	139	(86 – 189)	137	(67 – 189)
Diastolic blood pressure, <90 mmHg	83	(52 – 123)	81	(43 – 116)	80	(43 - 115)
Weight, kg	76	(39 – 115)	76	(38 - 117)	77	(42 - 120)

Imputation: LOFC or NOCB, as appropriate.
